# Impact of thrombus burden on long-term clinical outcomes in patients with either anterior or non-anterior ST-segment elevation myocardial infarction

**DOI:** 10.1007/s11239-021-02603-3

**Published:** 2021-11-26

**Authors:** Paola Scarparo, Menno van Gameren, Jeroen Wilschut, Joost Daemen, Wijnand K. Den Dekker, Felix Zijlstra, Nicolas M. Van Mieghem, Roberto Diletti

**Affiliations:** 1 Department of Interventional Cardiology, Thoraxcenter, Erasmus University Medical Center, Rotterdam, The Netherlands; 2 Amphia Hospital, Breda, The Netherlands

**Keywords:** ST-segment elevation myocardial infarction, Myocardial infarction, Anterior infarction, Thrombus burden, Percutaneous coronary intervention, Mortality

## Abstract

Large thrombus burden (LTB) during ST-segment elevation myocardial infarction (STEMI) could translate into worse clinical outcomes. The impact of a LTB in terms of long-term clinical outcomes on different myocardial infarct territories has not yet been fully evaluated. From April 2002 to December 2004, consecutive patients with STEMI undergoing percutaneous coronary intervention with drug eluting stent were evaluated. The study sample was stratified in two groups: anterior STEMI and non-anterior STEMI. LTB was considered as a thrombus larger than or equal to 2-vessel diameters, and small thrombus burden less than 2-vessel diameters. Major adverse cardiac events (MACE) were evaluated at 10-year and survival data were collected up to 15-year. A total of 812 patients were evaluated, 6 patients were excluded due to inadequate angiographic images, 410 (50.9%) had an anterior STEMI and 396 (49.1%) a non-anterior STEMI. Patients with LTB had higher rates of 10-year mortality (aHR 2.27, 95%CI 1.42–3.63; p = 0.001) and 10-year MACE (aHR 1.46, 95%CI 1.03–2.08; p = 0.033) in anterior STEMI, but not in non-anterior STEMI (aHR 0.78, 95%CI 0.49–1.24; p = 0.298; aHR 0.71, 95%CI 0.50–1.02; p = 0.062). LTB was associated with increased 30-day mortality (aHR 5.60, 95%CI 2.49–12.61; p < 0.001) and 30-day MACE (aHR 2.72, 95%CI 1.45–5.08; p = 0.002) in anterior STEMI, but not in non-anterior STEMI (aHR 0.39, 95%CI 0.15–1.06; p = 0.066; aHR 0.67, 95%CI 0.31–1.46; p = 0.316). Beyond 30-day, LTB had no impact on mortality and MACE in both groups. In anterior STEMI, LTB is associated with worse long-term clinical outcomes, this effect was driven by early events.

**Supplementary Information:**

The online version contains supplementary material available at 10.1007/s11239-021-02603-3.

## Highlights

- Large thrombus burden has a significant impact on mortality and MACE at 10 years in patients with anterior STEMI, but not in patients with non anterior STEMI.
- The impact of thrombus burden is mainly driven by early events.
- The reclassification of the thrombus burden after wire crossing in the occluded infarct related artery (G5) was applicable in almost every lesion, 99% of the cases.
- More than two thirds of the thrombotic occlusions, initially evaluated as large thrombus burden were actually caused by small thrombus.
- The reclassification of G5 might improve quantitative thrombus estimation.

## Introduction

Primary percutaneous coronary intervention (PCI) represents the gold standard therapy for coronary revascularization during ST-segment elevation myocardial infarction (STEMI) and a timely reperfusion strongly correlates with clinical outcomes [1]. However, despite the restoration of the epicardial coronary artery patency, the perfusion of the infarcted myocardium might be incomplete, due to microvascular obstruction and dysfunction [2].

The presence of large thrombus burden (LTB) in the infarct related artery (IRA) might increase the risk of distal embolization, microvascular obstruction, and no-reflow phenomenon leading to contractile dysfunction and irreversible myocardial damage [2–11].

Thrombus burden is classified by visual angiographic assessment in LTB defined as thrombus equal or greater than two vessel diameters and in small thrombus burden (STB) less than two vessel diameters [6]. In case of occluded IRA, thrombus burden is reclassified after guidewire crossing or small (diameter 1.5 mm) deflated balloon passage or dilation, as proposed by Sianos et al. [6].

Previous studies demonstrated that LTB is an independent predictor of early mortality, repeat myocardial infarction, and IRA revascularizations [6, 12].

In patients with STEMI, the anterior localization of the infarction is often associated with greater myocardial dysfunction, heart failure and increased mortality, mostly due to the larger myocardial territory supplied by the left anterior descending artery (LAD) [13–18].

Hypothetically, the embolization of a large amount of thrombotic debris in the territory supplied by LAD might lead to greater left ventricular dysfunction and worst prognosis, demanding more effective interventions.

The impact of LTB on clinical outcomes in different myocardial infarct territories has not yet been evaluated. Therefore, the purpose of the present study was to investigate the impact of the thrombus burden on very long-term clinical outcome in anterior and in non-anterior STEMI.

## Methods

### Study population

From April 2002 to December 2004, all consecutive patients with STEMI undergoing PCI in the Erasmus University Medical Center (EMC), Rotterdam, the Netherlands, were evaluated. Patients with STEMI treated with PCI and drug eluting stent (DES) within 12 h after the onset of myocardial ischemia symptoms were included in the analysis. Patients with non-quantifiable thrombus burden were excluded.

Demographic and clinical characteristics were assessed from the hospital database. All patients received a loading dose of aspirin and clopidogrel before the PCI procedure that was performed according to standard clinical practice. Thrombus aspiration and glycoprotein IIb/IIIa inhibitors (GPIs) treatment were at the operator’s discretion.

On the basis of IRA occlusion, the population was stratified into two groups: anterior STEMI due to the LAD occlusion and non-anterior STEMI due to non-LAD occlusion.

The Medical Ethics Committee of the EMC reviewed the study protocol and waived the need for additional informed consent because of the non-interventional character of this observational study using anonymous data collection. The investigation conforms to the principles outlined in the Declaration of Helsinki.

### Angiographic analysis

The angiographic data were revised by two experienced interventional cardiologists as described previously [6].

**Fig. 1:**
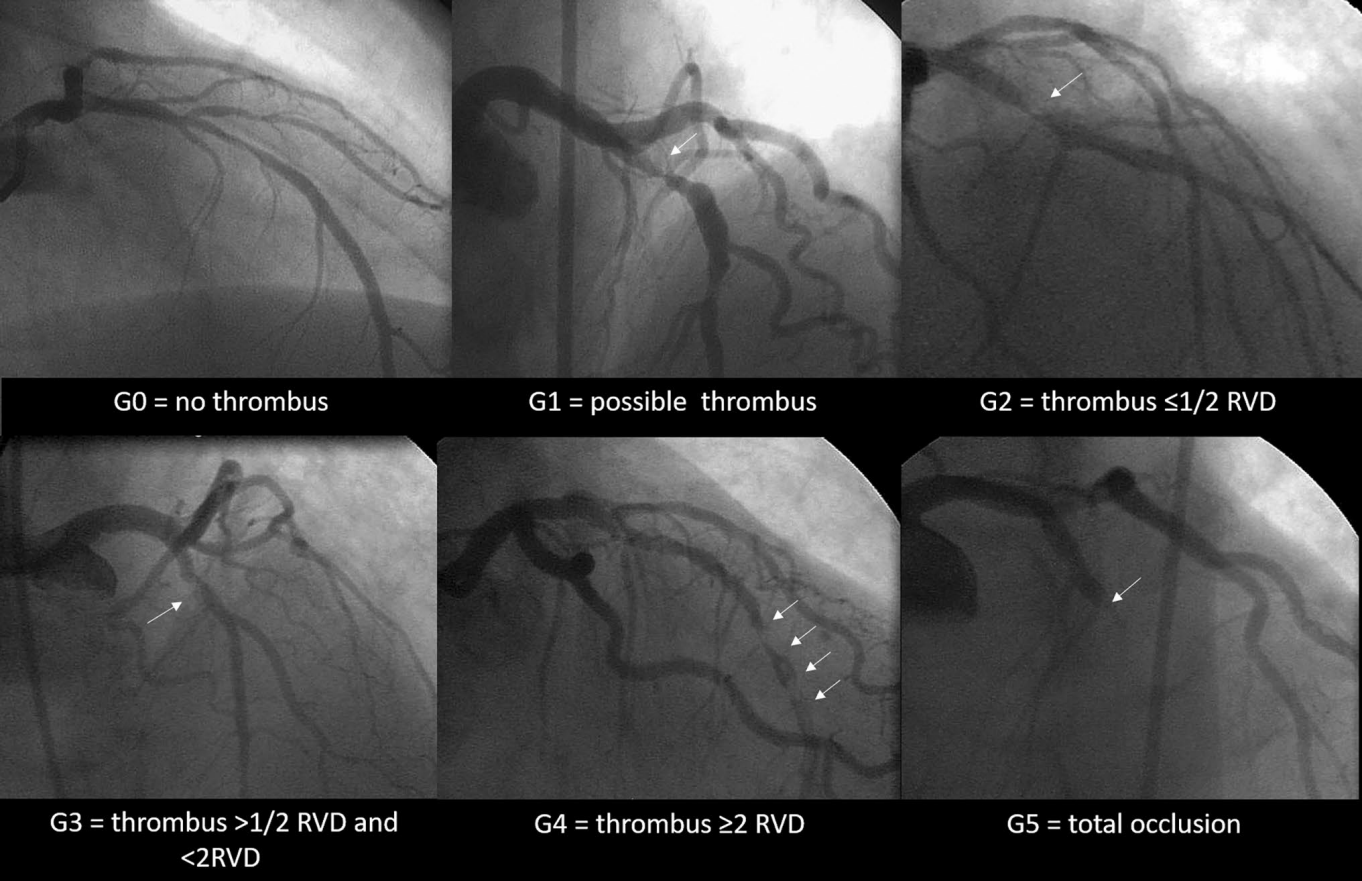
Thrombus burden classification. * RVD* reference vessel diameter. The Thrombolysis In the Myocardial Infarction (TIMI) thrombus classification consists of six grades from grade 0 (no thrombus) to grade 5 (total occlusion). Grade 5 represents an occluded infarct related artery and prevents thrombus size assessment. Grade 5 was reclassified after guidewire crossing or small (diameter 1.5 mm) deflated balloon passage. After G5 reclassification, large thrombus burden (LTB) was defined as being greater than or equal to two vessel diameters or greater (G4) and small thrombus burden (STB) defined by thrombus grade < G4

Intracoronary thrombus at baseline was angiographically identified and scored according to Thrombolysis in Myocardial Infarction (TIMI) thrombus grade [19]: grade 0 (G0) no angiographic characteristics of thrombus are present; grade 1 (G1) possible thrombus is present, with angiographic characteristics as reduced contrast density, haziness, irregular lesion contour, or a smooth convex meniscus, suggestive but not diagnostic of thrombus; grade 2 (G2) there is definite thrombus with greatest dimensions 1/2 or less of the vessel diameter; grade 3 (G3) there is definite thrombus with greatest linear dimension greater than 1/2 but less than 2 vessel diameters; grade 4 (G4) there is definite thrombus with the largest dimension at least 2 vessel diameters; grade 5 (G5) there is total occlusion (Fig. 1). In patients presenting with G5 in which the evaluation of the amount of thrombus was not possible, thrombus was reclassified into one of the other categories after flow achievement with either guidewire crossing, or a small (diameter 1.5 mm) deflated balloon passage or dilation [20]. After reclassification of the G5, thrombus burden was stratified in LTB with the largest dimension greater than or equal to two vessel diameters and in small thrombus burden (STB) with the largest dimension inferior than two vessel diameters.

No-reflow was defined as reduced anterograde flow (TIMI flow ≤ grade 2) in the absence of occlusion at the treatment site [21]. Distal embolization was defined as migration of a filling defect to distally occlude the IRA or one of its branches, or a new abrupt cut-off of the distal vessel or one of its branches [22].

### Clinical follow-up

The municipal civil registry in the Netherlands was consulted for the survival status of all patients.

Information on hospitalization and cardiovascular events were obtained through health questionnaires. If necessary, referring cardiologists and general practitioners were contacted for additional data. In case of re-hospitalization medical records or discharge letters from other hospitals were collected. Clinical follow-up was performed at 10 years and survival data were collected at 15 years.

Major adverse cardiac event (MACE) was defined as the composite of all-cause mortality, repeat myocardial infarction (MI), and target vessel revascularization (TVR). TVR was defined as any repeat percutaneous intervention or coronary artery bypass grafting of any segment of the infarct related artery.

### Statistical analysis

Continuous descriptive variables were expressed as mean ± standard deviation (SD) or median and interquartile range (IQR: 25th -75th percentile), and were compared using the Student’s *t*-test or Mann-Whitney U test as appropriate. Categorical variables expressed as numbers and percentages were compared by Pearson chi-square analysis or Fisher’s exact test, as appropriate.

Kaplan-Meier curves were generated for cumulative MACE and mortality events rates, the Cox proportional-hazard regression was used to establish the differences between groups.

The univariate analysis was performed using the Cox proportional hazards regression, with all the following variables: age, gender, diabetes mellitus, arterial hypertension, hypercholesterolemia, smoking, family history of coronary artery disease, previous myocardial infarction, previous PCI, primary PCI, stent thrombosis at the index procedure, cardiogenic shock, multivessel disease, multivessel PCI, baseline TIMI flow grade 0-1, glycoprotein IIb/IIIa inhibitors, bifurcation stenting, direct stenting, thrombectomy, final TIMI flow grade 3, no-reflow, distal embolization. Variables that were significant in univariate analysis at a level of p <0.10 were assessed in the multivariate Cox model. LTB was forced into the model to estimate its independent effect along with the other predictors of clinical outcomes.

Landmark analysis for mortality and MACE was performed with a 30-day landmark time point.

A 2-tailed p value of <0.05 was considered statistically significant and 95% confidence intervals (CI) were presented for all hazard ratio (HR). Statistical analyses were performed using the SPSS statistical software package for Windows, version 25.0 (IBM Corp., Armonk, New York).

## Results

Between April 2002 and December 2004, 812 consecutive patients with STEMI undergoing percutaneous revascularization with DES were evaluated. Six patients were excluded due to the inadequate angiographic images that made thrombus burden non-quantifiable, 806 patients were included in the analysis, 410 (50.9%) had an anterior STEMI and 396 (49.1%) had a non-anterior STEMI.

The baseline and angiographic characteristics of anterior and non-anterior STEMI according to thrombus burden are summarized in Table 1. The baseline and angiographic characteristics of the total population categorized by infarct location are summarized in Table S1 (Supplementary material). No significant differences were found between anterior STEMI and non-anterior STEMI in terms of demographics clinical confounders.

**Table 1:** Baseline and angiographic characteristics according to thrombus burden in anterior and non-anterior STEMI

Characteristic	Anterior STEMI			Non-anterior STEMI		
	STB (n = 304)	LTB (n = 106)	p-value	STB (n = 276)	LTB (n = 120)	p-value
Age (years)	59.1 ± 11.7	58.6 ± 12.2	0.664	59.0 ± 10.4	59.5 ± 11.6	0.732
Female	62 (20.4%)	18 (17.0%)	0.480	64 (23.2%)	27 (22.5%)	1
Diabetes mellitus	27 (8.9%)	14 (13.2%)	0.258	23 (8.3%)	16 (13.3%)	0.143
Arterial hypertension	76 (25.0%)	32 (30.2%)	0.307	74 (26.8%)	36 (30.0%)	0.543
Hypercholesterolemia	91 (29.9%)	28 (26.7%)	0.618	189 (68.7%)	85 (70.8%)	0.723
Smoking	115 (37.8%)	35 (33.0%)	0.413	113 (40.9%)	42 (35.0%)	0.313
Family history of CAD	79 (26.0%)	28 (26.4%)	1	70 (25.4%)	36 (30.0%)	0.387
Previous MI	25 (8.2%)	15 (14.2%)	0.088	30 (10.9%)	11 (9.2%)	0.721
Previous PCI	13 (4.3%)	11 (10.4%)	**0.029**	11 (0.4%)	11 (9.2%)	0.054
Previous CABG	1 (0.6%)	0 (0.0%)	0.588	4 (2.4%)	2 (3.5%)	0.646
MI presentation:						
Peak CK-MB (IU/l)	402.8 ± 416.0	326.6 ± 264.6	0.094	271.6 ± 200.7	274.4 ± 317.4	0.946
Primary PCI	274 (90.1%)	101 (95.3%)	0.111	241 (87.3%)	109 (90.8%)	0.394
Cardiogenic shock	23 (7.6%)	14 (13.2%)	0.113	28 (10.1%)	12 (10.0%)	1
Stent thrombosis	5 (1.6%)	9 (8.5%)	**0.002**	1 (0.4%)	7 (5.8%)	**0.001**
Multivessel coronary disease	108 (35.5%)	36 (34.0%)	0.814	124 (44.9%)	45 (37.5%)	0.186
Multivessel PCI	34 (11.2%)	9 (8.5%)	0.581	36 (13.0%)	7 (5.8%)	**0.035**
Inotropes	24 (7.9%)	14 (13.2%)	0.120	37 (13.4%)	17 (14.2%)	0.874
GP IIb/IIIa inhibitors	143 (47.0%)	70 (66.0%)	**0.001**	112 (40.6%)	81 (67.5%)	**<0.001**
Bifurcation stenting	24 (7.9%)	12 (11.3%)	0.319	10 (3.6%)	6 (5.0%)	0.581
Direct stenting	158 (52.0%)	55 (51.9%)	1	171 (62.0%)	66 (55.0%)	0.220
Thrombectomy	1 (0.3%)	29 (27.6%)	**<0.001**	3 (1.1%)	30 (25.0%)	**<0.001**
TIMI flow grade 0-1 at baseline	203 (66.8%)	96 (90.6%)	**<0.001**	189 (68.5%)	100 (83.3%)	**0.002**
Final TIMI flow grade3	242 (79.6%)	83 (79.0%)	0.889	213 (77.5%)	96 (80.0%)	0.599
No-reflow	1 (0.3%)	5 (4.7%)	**0.001**	2 (0.7%)	4 (3.3%)	0.071
Distal embolization	7 (2.3%)	12 (11.3%)	**<0.001**	13 (4.7%)	27 (22.5%)	**<0.001**

*CAD* coronary artery disease, *CK* creatine kinase, *GP* glycoprotein, *MI* myocardial infarction, *PCI* percutaneous coronary intervention, *LTB* large thrombus burden, *STB* small thrombus burden, *TIMI* thrombolysis in myocardial infarction

Higher peak of CK-MB, bifurcation stenting and distal embolization were higher in anterior STEMI than in non-anterior STEMI.

Multivessel disease and direct stenting were higher in non-anterior STEMI than in anterior STEMI.

### Angiographic classification of thrombus burden

**Fig. 2:**
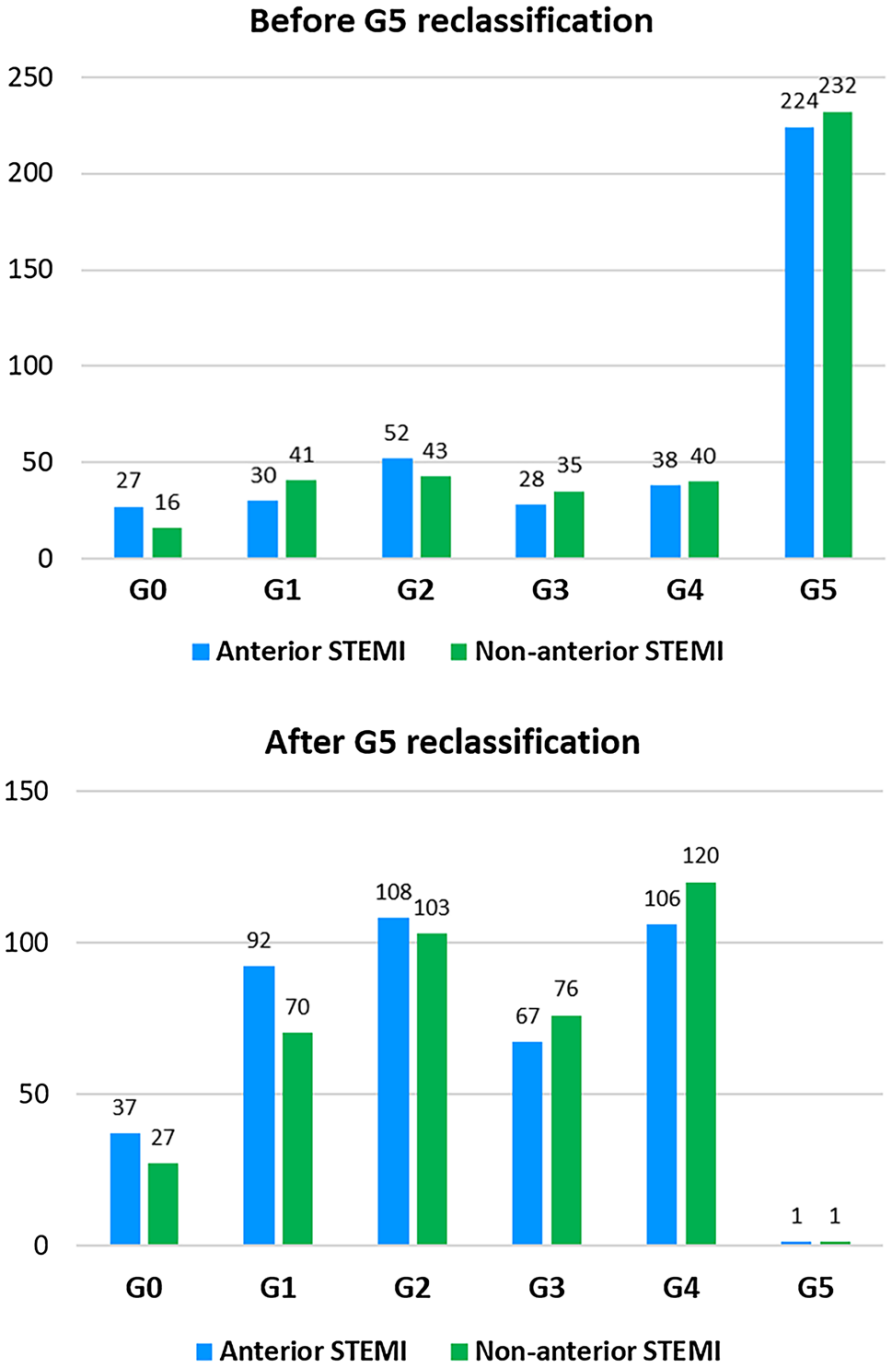
Distribution of thrombus burden in anterior STEMI and in non-anterior STEMI, before and after G5 reclassification. The distribution before G5 reclassification is based on the initial angiography. After G5 reclassification, patients who presented G5 at baseline were redistributed in the other categories after flow achievement

Out of the 806 patients more than half (56.6%, n = 456) presented an occluded IRA (G5) (Fig. 2). Reclassification of G5 was feasible in 454 (99.6%) patients, while in 2 (0.4%) no distal flow was achieved. After G5 reclassification, a total of 226 (28.0%) patients showed a LTB.

#### Follow-up events

Completeness of follow-up information at 10 years was obtained in 797 (98.9%) patients and survival data were recorded up to 15 years.

At 10-year, clinical outcomes were similar between patients with anterior and non-anterior STEMI in terms of mortality (24.9% vs. 28.4%; p = 0.297), MI (14.6% vs. 14.6%; p = 1), TVR (15.8% vs. 12.4%; p = 0.182) and overall MACE (41.1% vs. 43.7%; p = 0.474).

At 15-year, mortality rate was 34.8% (n = 277) without difference between anterior and non-anterior STEMI (33.7% vs. 35.8%; p = 0.552).

#### Before G5 reclassification analysis

**Fig. 3:**
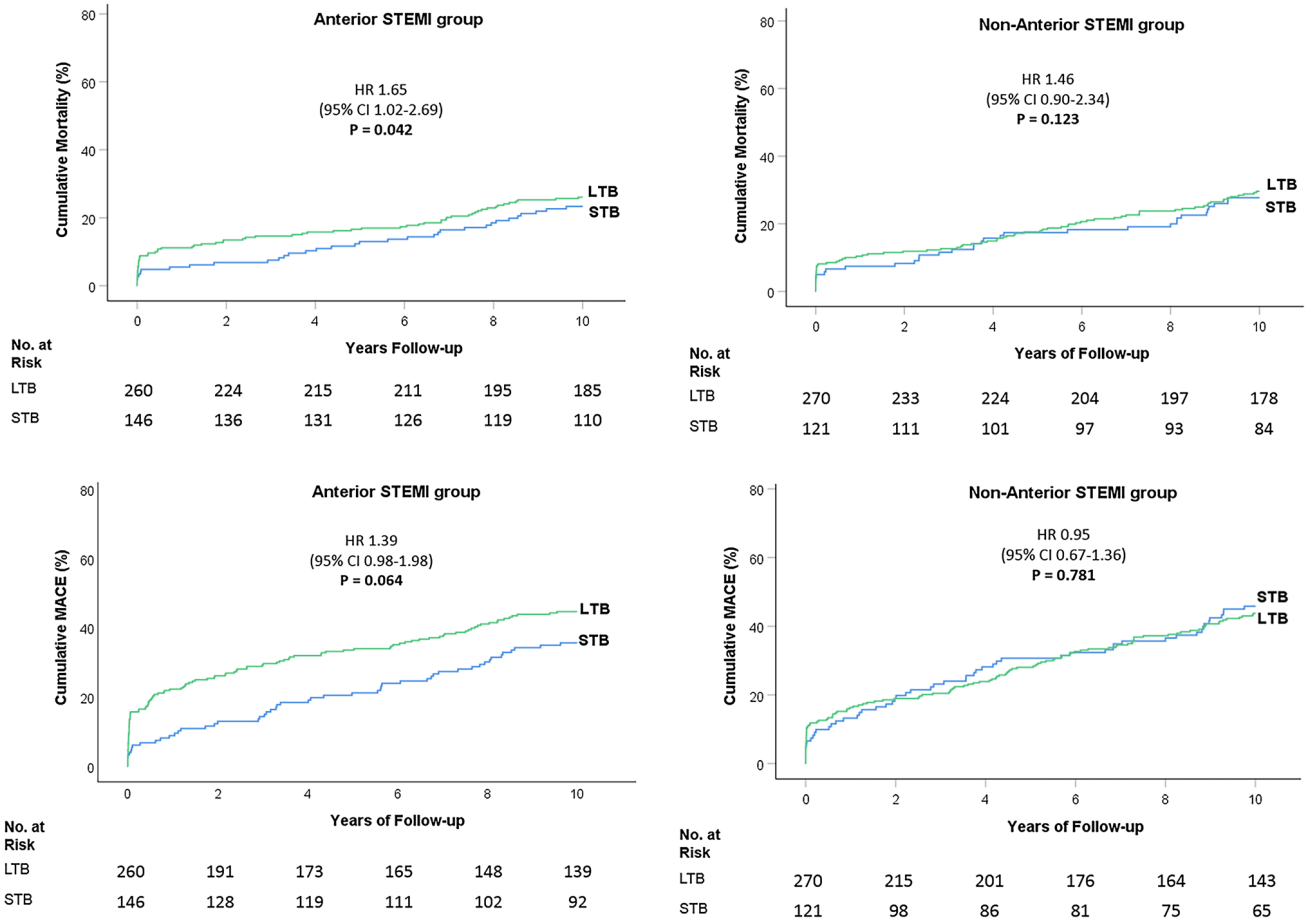
Ten-year cumulative Mortality and MACE according to the Infarct-Related Artery Thrombus Burden before G5 reclassification. * LTB* large thrombus burden, *STB* small thrombus burden. Kaplan-Meier curves for 10-year mortality and MACE (death, repeat non-fatal myocardial infarction, or target vessel revascularization) rates before G5 reclassification

In anterior STEMI, before G5 reclassification, 10-year mortality rate was higher in LTB than in STB (25.8% vs. 23.3%; aHR 1.65, 95%CI 1.02–2.69; p = 0.042), and 10-year MACE rate was similar between the two groups (44.2% vs. 35.6%; aHR 1.39, 95%CI 0.98–1.98; p = 0.064) (Fig. 3).

In non-anterior STEMI, before G5 reclassification, 10-year mortality and 10-year MACE were similar between the two groups (mortality: 28.9% vs. 27.3%; aHR 1.46, 95%CI 0.90–2.34; p = 0.123 and MACE 43.0% vs. 45.5%; aHR 0.95, 95%CI 0.67–1.36; p = 0.781) (Fig. 3).

#### After G5 reclassification analysis

**Fig. 4:**
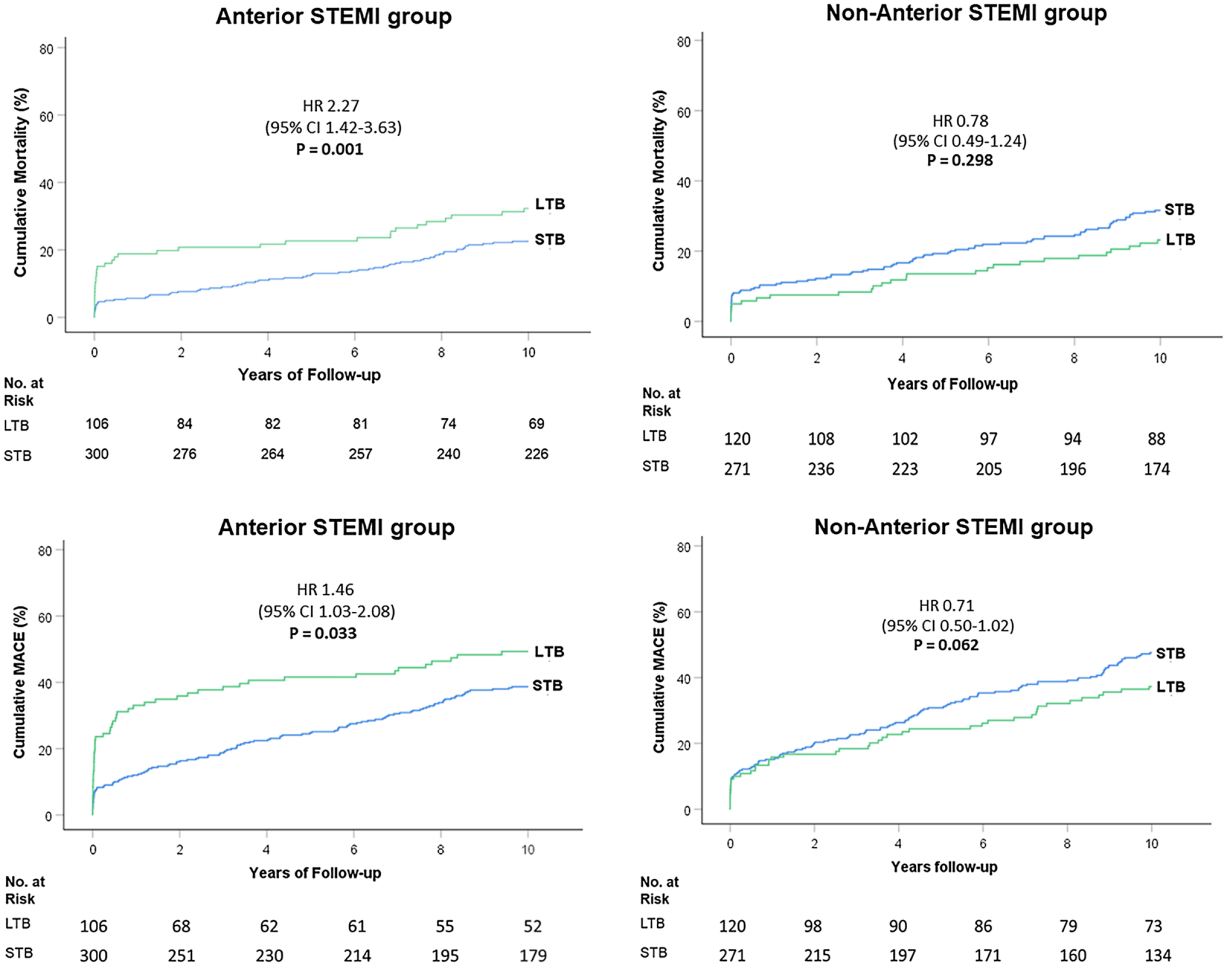
Ten-year cumulative Mortality and MACE according to the Infarct-Related Artery Thrombus Burden after G5 reclassification. * LTB* large thrombus burden, *STB* small thrombus burden. Kaplan-Meier curves for 10-year mortality and MACE (death, repeat non-fatal myocardial infarction, or target vessel revascularization) rates after G5 reclassification

At 10 years, increased mortality and MACE rates occurred among patients with LTB compared with STB (mortality: 32.1% vs. 22.3%; aHR 2.27, 95%CI 1.42–3.63; p = 0.001, and MACE: 49.1% vs. 38.3%; aHR 1.46, 95%CI 1.03–2.08; p = 0.033) (Table S2 and Table S3 Supplementary Material) in the anterior STEMI group, but no significant differences were found in the non-anterior STEMI group (mortality: 22.5% vs. 31.0%; aHR 0.78, 95%CI 0.49–1.24; p = 0.298, and MACE: 36.7% vs. 46.7%; aHR 0.71, 95%CI 0.50–1.02; p = 0.062) (Fig. 4) (Tables 2 and 3).

**Table 2:** Clinical Outcomes in patients with large thrombus burden and small thrombus burden in Anterior STEMI

	LTB (n = 106 )	STB (n = 304)	HR (95%CI)	p value	aHR (95%CI)	p value
10-year						
Mortality	32.1%	22.3%	1.60 (1.06-2.42)	0.026	2.27 (1.42-3.63)	**0.001**
MACE	49.1%	38.3%	1.55 (1.12-2.15)	0.009	1.46 (1.03-2.08)	**0.033**
30-day						
Mortality	15.1%	4.3%	1.06 (1.79-7.72)	<0.001	5.60 (2.49-12.61)	**<0.001**
MACE	23.6%	7.7%	3.31 (1.88-5.84)	<0.001	2.72 (1.45-5.08)	**0.002**
After 30-day						
Mortality	17.0%	18.3%	1.06 (0.62-1.80)	0.842	1.24 (0.43-1.11)	0.484
MACE	33.3%	33.2%	1.06 (0.69-1.62)	0.806	1.01 (0.65-1.58)	0.967

*aHR* adjusted hazard ratio, *CI* confidence interval, *HR* hazard ratio, *LTB* large thrombus burden, MACE: *STB* small thrombus burden, *MACE* major adverse cardiac events

**Table 3:** Clinical outcomes in patients with large thrombus burden and small thrombus burden in non-anterior STEMI

	LTB (n = 120)	STB (n = 276)	HR (95%CI)	p value	aHR (95%CI)	p value
10-year						
Mortality	22.5%	31.3%	0.69 (0.45-1.07)	0.095	0.78 (0.49-1.24)	0.298
MACE	36.7%	46.7%	0.74 (0.53-1.04)	0.086	0.71 (0.50-1.02)	0.062
30-day						
Mortality	5.0%	8.3%	0.59 (0.24-1.45)	0.252	0.39 (0.15-1.06)	0.066
MACE	9.2%	10.0%	0.92 (0.46-1.85)	0.815	0.67 (0.31-1.46)	0.316
After 30-day						
Mortality	17.5%	22.9%	0.72 (0.44-1.18)	0.191	0.87 (0.52-1.43)	0.574
MACE	30.3%	41.2%	0.70 (0.47-1.03)	0.070	0.76 (0.51-1.13)	0.169

*aHR* adjusted hazard ratio, *CI* confidence interval, *HR* hazard ratio, *LTB* large thrombus burden, MACE: *STB* small thrombus burden, *MACE* major adverse cardiac events

In the anterior STEMI group, the landmark survival analysis demonstrated a higher 30-day mortality rate in LTB than in STB (15.1% vs. 4.3%; aHR 5.60, 95%CI 2.49–12.61; p < 0.001). Beyond 30-day, morality rate was similar between the two groups (LTB 17.0% vs. STB 18.3%; aHR 1.24, 95%CI 0.43–1.11; p = 0.484) (Table 2, Table S4 and Table S5 Supplementary Material).

In the anterior STEMI group, the landmark analysis at 30 days shown a higher MACE rate in patients with LTB (LTB 23.6% vs. STB 7.7%; aHR 2.72, 95%CI 1.45–5.08; p = 0.002). Thereafter, MACE rates were comparable between patients with LTB and STB (33.3% vs. 33.2%; aHR 1.01, 95%CI 0.65–1.58; p = 0.967) (Table 2).

**Fig. 5:**
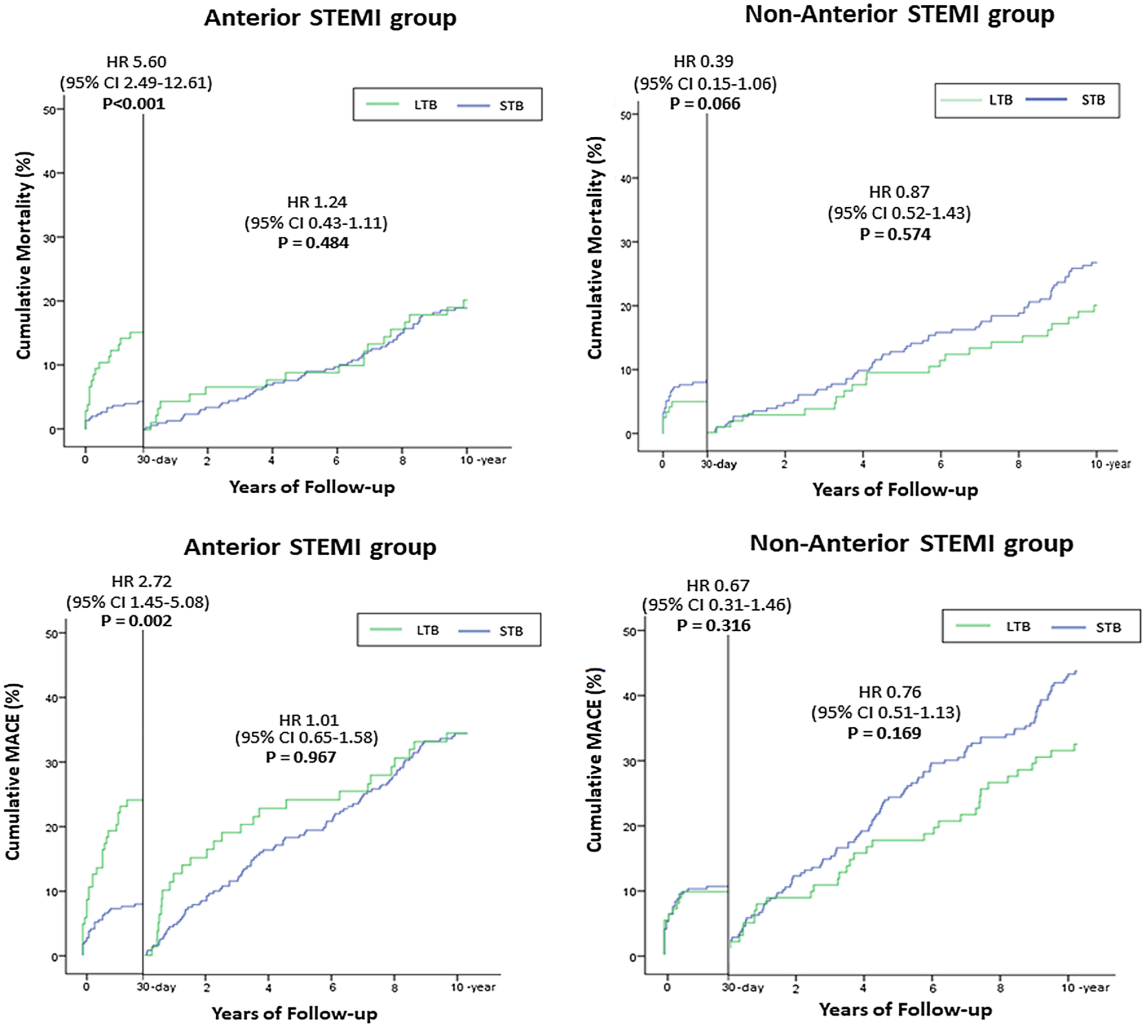
Landmark analysis at 30-day for Mortality and MACE according to thrombus burden in anterior and non-anterior STEMI. * LTB* large thrombus burden, *STB* small thrombus burden. Landmark analysis for mortality and MACE with a 30-day landmark time point in anterior STEMI and non-anterior STEMI

In the non-anterior STEMI group, mortality was similar between LTB and STB at 30-day (LTB 5.0% vs. STB 8.3%; aHR 0.39, 95%CI 0.15–1.06; p = 0.066) and beyond 30-day (LTB 17.5% vs. STB 22.9%; aHR 0.87, 95%CI 0.52–1.43; p = 0.574) (Fig. 5) (Table 3).

In non-anterior STEMI, the landmark analysis indicated thrombus burden had no impact on MACE at 30 days (LTB 9.2% vs. STB 10.0%; aHR 0.67, 95%CI 0.31–1.46; p = 0.316) and neither after 30 days (LTB 30.3% vs. STB 41.2%; aHR 0.76, 95%CI 0.51–1.13; p = 0.169) (Fig. 5) (Table 3).

## Discussion

The present study investigated the impact of LTB on very long-term clinical outcomes in anterior and non-anterior STEMI. The major findings are (1) LTB had a significant impact on mortality and MACE at 10 years in the anterior STEMI group, but not in the non-anterior STEMI group, (2) the effect of LTB was mainly driven by early events (≤30 days), after 30 days the thrombus burden had a limited impact on clinical outcomes, and (3) the reclassification of G5 might improve quantitative thrombus estimation, when thrombus burden was reclassified, differences in MACE rate became evident between the groups.

An adequate evaluation of the real amount of the thrombus burden is essential to identify the lesions that might be at high risk of distal embolization, in particular the total occlusion of the infarct related artery, namely thrombus grade G5, reflects only the state of the flow rather than the real amount of the thrombus.

In our analysis the reclassification of G5 after wire or small balloon passage was applicable in almost every (99.6%) lesion. More than two thirds (67.5%) of the thrombotic occlusions (G5), initially evaluated as large thrombus burden were actually caused by small thrombus.

In anterior STEMI, mortality and MACE rates were higher in patients with LTB compared with those with STB; conversely, the amount of thrombus had a negligible effect on clinical outcomes in non-anterior STEMI.

These findings might be related to the higher myocardial mass perfused by the LAD compared to other myocardial regions [23]. Usually, infarcts caused by LAD occlusion are associated with a larger left ventricular damage and an increased risk of heart failure and death [17], this may be due to the larger infarct size rather than the mere infarct localization. In a recent patients level pooled analysis of ten randomized trials, a strong association between infarct size and all-cause mortality was demonstrated regardless of the infarct location, although the anterior infarct location was a strong determinant of increased infarct size [18].

On the other hand, in non-anterior infarctions, distal embolization might play a less relevant role on infarct size given the smaller area perfused by the right coronary artery or by the circumflex.

The present study is the first reporting very long term clinical outcomes in patients with acute myocardial infarction stratified per thrombus burden and infarct localization. In our analysis, the impact of thrombus burden in anterior STEMI was mainly driven by the early events and beyond 30 days, up to 10-year, thrombus burden was poorly associated with mortality and MACE.

Given our results, patients presenting with anterior STEMI and large thrombus burden may represent a sub-population at high risk of distal embolization and higher short-term event rate, which might benefit by additional periprocedural therapeutic strategies.

Current European and American guidelines on myocardial revascularization in patients with STEMI suggest glycoprotein IIb/IIIa antagonist and/or thrombectomy as for bail-out scenarios including high thrombus burden or thrombotic complications, although specific studies are lacking [1, 24, 25]. In a recent meta-analysis from three randomized trials on thrombus aspiration, TAPAS, TASTE, and TOTAL trials, patients with high thrombus burden treated with manual thrombectomy had a reduced cardiovascular mortality but increased cerebrovascular events at 30 days compared to those treated with PCI only [8, 26–28]. In addition, in TAPAS and TOTAL trials thrombus grade was assessed only before wire crossing, a thrombus grade reclassification in cases with thrombus burden G5, might have unveiled even more evident differences between groups. [26–30].

In our analysis a large thrombus burden was associated with increased mortality and MACE mainly during the first month after primary PCI for anterior infarction, suggesting that a highly thrombotic milieu might affect in particular procedural complication and early events. In this scenario not only thrombus aspiration but also additional pharmacologic strategies to achieve rapid platelet inhibition could be considered to minimize the negative impact of large thrombus burden in anterior STEMI [31–34].

## Limitations

This is a single center, observational, retrospective study with its inherent limitations of selection bias and missing data. A small percentage of the population underwent rescue PCI and thrombus burden modification cannot be excluded. Only first-generation DES were implanted. Finally antiplatelet therapy was prescribed for at least 6 months according to recommendations at that time.

Further technologies and development of thrombus aspiration devices are warranted to improve the effectiveness of thrombus removal without increase the risk of stroke; thrombectomy may particularly benefit patients with a large thrombotic burden.

## Conclusions

Large thrombus burden is associated with higher mortality and MACE rate in patients with anterior STEMI but not in non-anterior STEMI. The impact of a large thrombus burden on clinical outcomes is mainly associated with early events.

## Supplementary Information

Below is the link to the electronic supplementary material.

Supplementary material 1 (DOCX 27.9 kb)

## Data Availability

Data available on request from the authors.
