# Supplementary material for: Impact of thrombus burden on long-term clinical outcomes in patients with either anterior or non-anterior ST-segment elevation myocardial infarction
Source: J Thromb Thrombolysis. 2021 Nov 26;54(1):47–57. doi: 10.1007/s11239-021-02603-3 (PMC9259523; doi:10.1007/s11239-021-02603-3)
Supplement: Supplementary file 1 — Supplementary material 1 (DOCX 27.9 kb) [file 11239_2021_2603_MOESM1_ESM.docx]

**Supplementary Material**

**Table S1. Baseline Clinical and Angiographic characteristics**

| **Characteristic** | **Total**  **(n=806)** | **Anterior STEMI**  **(n=410)** | **Non-anterior**  **STEMI**  **(n=396)** | **p value** |
| --- | --- | --- | --- | --- |
| Age (years) | 59.1±11.5 | 58.9±11.8 | 59.3±11.2 | 0.663 |
| Female | 171 (21.2%) | 80 (19.5%) | 91 (23.0%) | 0.263 |
| Diabetes mellitus | 80 (9.9%) | 41 (10.0%) | 39 (9.8%) | 1.000 |
| Arterial hypertension | 218 (27.0%) | 108 (26.3%) | 110 (27.8%) | 0.692 |
| Hypercholesterolemia | 240 (29.9%) | 119 (29.1%) | 121 (30.6%) | 0.644 |
| Smoking | 305 (37.8%) | 150 (36.6%) | 155 (39.1%) | 0.468 |
| Family history of CAD | 213 (26.4%) | 107 (26.1%) | 106 (26.8%) | 0.873 |
| Previous MI | 81 (10.0%) | 40 (9.8%) | 41 (10.4%) | 0.815 |
| Previous PCI | 46 (5.7%) | 24 (5.9%) | 22 (5.6%) | 0.880 |
| Previous CABG | 7 (1.6%) | 1 (0.5%) | 6 (2.7%) | 0.123 |
| MI presentation |  |  |  |  |
| - Infarct duration (h) | 4.7±11.2 | 3.3±3.7 | 5.8±16.0 | 0.075 |
| - Peak CK-MB (IU/l) | 312.7±301.6 | 346.9±313.2 | 273.5±283.4 | **0.009** |
| - Primary PCI | 725 (90.0%) | 375 (91.5%) | 350 (88.4%) | 0.160 |
| - Cardiogenic shock | 77 (9.6%) | 37 (9.0%) | 40 (10.1%) | 0.633 |
| - Stent thrombosis | 22 (2.7%) | 14 (3.4%) | 8 (2.0%) | 0.281 |
| Multivessel coronary disease | 313 (38.8%) | 144 (35.1%) | 169 (42.7%) | **0.030** |
| Multivessel PCI | 86 (10.7%) | 43 (10.5%) | 43 (10.9%) | 0.909 |
| Inotropes | 92 (11.4%) | 38 (9.3%) | 54 (13.6%) | 0.059 |
| Glycoprotein IIb/IIIa inhibitors | 406 (50.4%) | 213 (52.0%) | 193 (48.7%) | 0.398 |
| Bifurcation stenting | 52 (6.5%) | 36 (8.8%) | 16 (4.0%) | **0.006** |
| Direct stenting | 450 (55.8%) | 213 (52.0%) | 237 (59.8%) | **0.028** |
| Thrombectomy | 63 (7.8%) | 30 (7.3%) | 33 (8.4%) | 0.602 |
| TIMI flow at baseline |  |  |  |  |
| - 0 | 464 (57.6%) | 228 (55.6%) | 236 (59.6%) | 0.25 |
| - 1 | 124 (15.4%) | 71(17.3%) | 53 (13.4%) | 0.14 |
| - 2 | 112 (13.9%) | 62 (15.1%) | 50 (12.6%) | 0.31 |
| - 3 | 106 (13.2%) | 49 (12.0%) | 57 (14.4%) | 0.34 |
| TIMI flow grade 0-1 | 588 (73%) | 299 (72.9%) | 289 (73.0%) | 1.000 |
| Final TIMI flow grade 3 | 634 (78.9%) | 325 (79.5%) | 309 (78.2%) | 0.730 |
| No-reflow | 12 (1.5%) | 6 (1.5%) | 6 (1.5%) | 1.000 |
| Distal embolization | 59 (7.3%) | 19 (4.6%) | 40 (10.1%) | **0.004** |
| LTB (before reclassification) | 534 (66.3%) | 262 (63.9%) | 272 (66.3%) | 0.157 |
| LTB (after reclassification) | 226 (28.0%) | 106 (25.9%) | 120 (30.3%) | 0.182 |

CAD: coronary artery disease; CK: creatine kinase; MI: myocardial infarction; PCI: percutaneous coronary intervention; LTB: large thrombus burden; TIMI: Thrombolysis In Myocardial Infarction.

**Table S2. Predictors of 10-year mortality in patients with anterior STEMI**

|  | **Unadjusted** | | | **Adjusted** | | |
| --- | --- | --- | --- | --- | --- | --- |
|  | **HR** | **95% CI** | **p value** | **HR** | **95% CI** | **p value** |
| LTB | 1.60 | 1.06-2.42 | 0.026 | 2.27 | 1.42-3.63 | **0.001** |
| Age (years) | 1.07 | 1.04-1.09 | <0.001 | 1.06 | 1.04-1.08 | **<0.001** |
| Female | 1.41 | 0.89-2.24 | 0.138 |  |  |  |
| Diabetes mellitus | 1.31 | 0.72-2.39 | 0.379 |  |  |  |
| Arterial hypertension | 0.96 | 0.62-1.50 | 0.865 |  |  |  |
| Hypercholesterolemia | 2.07 | 1.24-3.45 | 0.005 | 1.78 | 1.05-3.02 | **0.031** |
| Smoking | 0.61 | 0.40-0.95 | 0.028 | 1.30 | 0.80-2.11 | 0.300 |
| Family history of CAD | 0.24 | 0.12-0.48 | <0.001 | 0.33 | 0.16-0.70 | **0.004** |
| Previous MI | 2.26 | 1.36-3.77 | 0.002 | 1.27 | 0.72-2.26 | 0.412 |
| Previous PCI | 1.42 | 0.69-2.92 | 0.345 |  |  |  |
| Primary PCI | 1.92 | 0.78-4.72 | 0.154 |  |  |  |
| Stent thrombosis at index procedure | 1.94 | 0.85-4.42 | 0.117 |  |  |  |
| Cardiogenic shock | 3.17 | 1.91-5.29 | <0.001 | 2.37 | 1.36-4.14 | **0.002** |
| Multivessel disease | 1.99 | 1.35-2.94 | 0.001 | 1.65 | 1.08-2.52 | **0.020** |
| Multivessel PCI | 1.43 | 0.81-2.51 | 0.215 |  |  |  |
| Baseline TIMI flow grade 0-1 | 0.94 | 0.61-1.44 | 0.766 |  |  |  |
| Glycoprotein IIb/IIIa inhibitors | 0.54 | 0.37-0.81 | 0.003 | 0.51 | 0.33-0.78 | **0.002** |
| Bifurcation stenting | 0.92 | 0.45-1.89 | 0.814 |  |  |  |
| Direct stenting | 0.69 | 0.47-1.02 | 0.061 | 0.80 | 0.53-1.21 | 0.286 |
| Thrombectomy | 0.94 | 0.45-2.03 | 0.873 |  |  |  |
| Final TIMI flow grade 3 | 0.68 | 0.44-1.07 | 0.094 | 0.86 | 0.52-1.41 | 0.536 |
| No reflow | 3.29 | 1.04-10.39 | 0.042 | 1.20 | 0.32-4.47 | 0.784 |
| Distal embolization | 1.15 | 0.47-2.83 | 0.760 |  |  |  |

HR, hazard ratio; CI confidence interval; LTB, large thrombus burden; CAD, coronary artery disease; MI, myocardial infarction; PCI, percutaneous coronary intervention; TIMI, Thrombolysis In Myocardial Infarction.

Results of the univariate logistic regression analyses investigating 23 variables as potential predictors of ten-year mortality incidence and of the multivariate analysis using the 12 variables significant at p≤0.10 in the univariate analysis.

**Table S3. Predictors of 10-year MACE in patients with anterior STEMI**

|  | **Unadjusted** | | | **Adjusted** | | |
| --- | --- | --- | --- | --- | --- | --- |
|  | **HR** | **95% CI** | **p value** | **HR** | **95% CI** | **p value** |
| LTB | 1.55 | 1.12-2.15 | 0.009 | 1.46 | 1.03-2.08 | **0.033** |
| Age (years) | 1.02 | 1.01-1.03 | 0.002 | 1.01 | 1.00-1.03 | **0.041** |
| Female | 1.30 | 0.90-1.88 | 0.158 |  |  |  |
| Diabetes mellitus | 1.29 | 0.80-2.07 | 0.301 |  |  |  |
| Arterial hypertension | 0.99 | 0.70-1.40 | 0.937 |  |  |  |
| Hypercholesterolemia | 1.60 | 1.11-2.30 | 0.012 | 1.61 | 1.10-2.34 | **0.013** |
| Smoking | 0.83 | 0.61-1.15 | 0.267 |  |  |  |
| Family history of CAD | 0.51 | 0.34-0.77 | 0.001 | 0.62 | 0.40-0.96 | **0.030** |
| Previous MI | 2.80 | 1.88-4.16 | <0.001 | 2.18 | 1.33-3.58 | **0.002** |
| Previous PCI | 2.76 | 1.67-4.57 | <0.001 | 1.47 | 0.57-3.77 | 0.422 |
| Primary PCI | 0.53 | 0.27-1.03 | 0.061 | 0.45 | 0.23-0.91 | **0.026** |
| Stent thrombosis at index procedure | 3.21 | 1.74-5.94 | <0.001 | 1.21 | 0.41-3.60 | 0.732 |
| Cardiogenic shock | 1.87 | 1.17-2.98 | 0.009 | 1.49 | 0.89-2.47 | 0.126 |
| Multivessel disease | 1.44 | 1.06-1.97 | 0.020 | 1.29 | 0.93-1.80 | 0.129 |
| Multivessel PCI | 0.88 | 0.53-1.48 | 0.632 |  |  |  |
| Baseline TIMI flow grade 0-1 | 1.14 | 0.80-1.61 | 0.467 |  |  |  |
| Glycoprotein IIb/IIIa inhibitors | 1.00 | 0.74-1.36 | 0.994 |  |  |  |
| Bifurcation stenting | 1.38 | 0.84-2.28 | 0.209 |  |  |  |
| Direct stenting | 0.68 | 0.50-0.92 | 0.013 | 0.87 | 0.63-1.19 | 0.377 |
| Thrombectomy | 0.79 | 0.42-1.49 | 0.464 |  |  |  |
| Final TIMI flow grade 3 | 0.75 | 0.53-1.07 | 0.112 |  |  |  |
| No reflow | 1.78 | 0.57-5.58 | 0.322 |  |  |  |
| Distal embolization | 1.60 | 0.84-3.03 | 0.152 |  |  |  |

HR, hazard ratio; CI confidence interval; LTB, large thrombus burden; CAD, coronary artery disease; MI, myocardial infarction; PCI, percutaneous coronary intervention; TIMI, Thrombolysis In Myocardial Infarction.

Results of the univariate logistic regression analyses investigating 23 variables as potential predictors of ten-year MACE incidence and of the multivariate analysis using the 11 variables significant at p≤0.10 in the univariate analysis.

**Table S4. Predictors of 30-day mortality in patients with anterior STEMI**

|  | **Unadjusted** | | | **Adjusted** | | |
| --- | --- | --- | --- | --- | --- | --- |
|  | **HR** | **95% CI** | **p value** | **HR** | **95% CI** | **p value** |
| LTB | 3.71 | 1.79-7.72 | <0.001 | 5.60 | 2.49-12.61 | **<0.001** |
| Age (years) | 1.06 | 1.03-1.20 | 0.001 | 1.03 | 0.99-1.07 | 0.087 |
| Female | 1.91 | 0.87-4.20 | 0.107 |  |  |  |
| Diabetes mellitus | 1.47 | 0.51-4.24 | 0.471 |  |  |  |
| Arterial hypertension | 1.25 | 0.57-2.74 | 0.584 |  |  |  |
| Hypercholesterolemia | 0.17 | 0.41-0.73 | 0.017 | 4.82 | 1.13-20.49 | **0.033** |
| Smoking | 0.35 | 0.13-0.91 | 0.031 | 0.93 | 0.32-2.68 | 0.890 |
| Family history of CAD | 0.20 | 0.04-0.85 | 0.030 | 0.32 | 0.07-1.42 | 0.133 |
| Previous MI | 2.04 | 0.78-5.34 | 0.148 |  |  |  |
| Previous PCI | 0.57 | 0.08-4.21 | 0.584 |  |  |  |
| Primary PCI | 0.78 | 0.19-3.29 | 0.738 |  |  |  |
| Stent thrombosis at index procedure | 1.03 | 0.14-7.59 | 0.975 |  |  |  |
| Cardiogenic shock | 8.97 | 4.27-18.80 | <0.001 | 6.36 | 2.76-14.68 | **<0.001** |
| Multivessel disease | 2.04 | 0.98-4.22 | 0.056 | 1.68 | 0.74-3.80 | 0.212 |
| Multivessel PCI | 1.38 | 0.48-3.98 | 0.546 |  |  |  |
| Baseline TIMI flow grade 0-1 | 1.80 | 0.69-4.72 | 0.232 |  |  |  |
| Glycoprotein IIb/IIIa inhibitors | 0.34 | 0.15-0.77 | 0.010 | 0.26 | 0.10-0.63 | **0.003** |
| Bifurcation stenting | 2.22 | 0.85-5.82 | 0.105 |  |  |  |
| Direct stenting | 0.65 | 0.31-1.37 | 0.256 |  |  |  |
| Thrombectomy | 0.95 | 0.23-3.98 | 0.947 |  |  |  |
| Final TIMI flow grade 3 | 0.55 | 0.25-1.12 | 0.55 |  |  |  |
| No reflow | 0.55 | 0.25-1.21 | 0.140 |  |  |  |
| Distal embolization | 1.58 | 0.38-6.62 | 0.535 |  |  |  |

HR, hazard ratio; CI confidence interval; LTB, large thrombus burden; CAD, coronary artery disease; MI, myocardial infarction; PCI, percutaneous coronary intervention; TIMI, Thrombolysis In Myocardial Infarction.

Results of the univariate logistic regression analyses investigating 23 variables as potential predictors of 30-day mortality incidence and of the multivariate analysis using the 8 variables significant at p≤0.10 in the univariate analysis.

**Table S5. Predictors of 30-day MACE in patients with anterior STEMI**

|  | **Unadjusted** | | | **Adjusted** | | |
| --- | --- | --- | --- | --- | --- | --- |
|  | **HR** | **95% CI** | **p value** | **HR** | **95% CI** | **p value** |
| LTB | 3.31 | 1.88-5.84 | <0.001 | 2.72 | 1.45-5.08 | **0.002** |
| Age (years) | 1.02 | 0.99-1.05 | 0.075 | 1.02 | 0.99-1.05 | 0.150 |
| Female | 1.61 | 0.85-3.04 | 0.144 |  |  |  |
| Diabetes mellitus | 1.61 | 0.72-3.59 | 0.243 |  |  |  |
| Arterial hypertension | 1.37 | 0.75-2.49 | 0.309 |  |  |  |
| Hypercholesterolemia | 1.82 | 0.88-3.76 | 0.107 |  |  |  |
| Smoking | 0.69 | 0.37-1.28 | 0.233 |  |  |  |
| Family history of CAD | 0.62 | 0.30-1.29 | 0.202 |  |  |  |
| Previous MI | 2.64 | 1.32-5.30 | 0.006 | 1.34 | 0.59-3.02 | 0.484 |
| Previous PCI | 2.46 | 1.05-5.79 | 0.039 | 1.60 | 0.60-4.27 | 0.347 |
| Primary PCI | 0.44 | 0.11-1.83 | 0.262 |  |  |  |
| Stent thrombosis at index procedure | 1.96 | 0.61-6.30 | 0.260 |  |  |  |
| Cardiogenic shock | 4.14 | 2.15-7.97 | <0.001 | 2.51 | 1.21-5.23 | **0.014** |
| Multivessel disease | 2.51 | 1.42-4.43 | 0.002 | 1.83 | 0.99-3.38 | 0.056 |
| Multivessel PCI | 1.00 | 0.40-2.53 | 0.997 |  |  |  |
| Baseline TIMI flow grade 0-1 | 1.93 | 0.90-4.12 | 0.091 | 1.34 | 0.60-3.02 | 0.479 |
| Glycoprotein IIb/IIIa inhibitors | 0.92 | 0.52-1.61 | 0.762 |  |  |  |
| Bifurcation stenting | 2.52 | 1.22-5.21 | 0.012 | 1.89 | 0.88-4.03 | 0.102 |
| Direct stenting | 0.70 | 0.40-1.25 | 0.228 |  |  |  |
| Thrombectomy | 0.86 | 0.27-2.76 | 0.794 |  |  |  |
| Final TIMI flow grade 3 | 0.83 | 0.42-1.63 | 0.584 |  |  |  |
| No reflow | 6.74 | 2.09-21.75 | 0.001 | 2.08 | 0.56-7.75 | 0.276 |
| Distal embolization | 1.95 | 0.70-5.42 | 0.202 |  |  |  |

HR, hazard ratio; CI confidence interval; LTB, large thrombus burden; CAD, coronary artery disease; MI, myocardial infarction; PCI, percutaneous coronary intervention; TIMI, Thrombolysis In Myocardial Infarction.

Results of the univariate logistic regression analyses investigating 23 variables as potential predictors of 30-day MACE incidence and of the multivariate analysis using the 9 variables significant at p≤0.10 in the univariate analysis.
